# Risk Factors and Mouse Models of Abdominal Aortic Aneurysm Rupture

**DOI:** 10.3390/ijms21197250

**Published:** 2020-09-30

**Authors:** Smriti Murali Krishna, Susan K. Morton, Jiaze Li, Jonathan Golledge

**Affiliations:** 1 The Vascular Biology Unit, Queensland Research Centre for Peripheral Vascular Disease, College of Medicine and Dentistry, James Cook University, Townsville, QLD 4811, Australia; smriti.krishna@jcu.edu.au (S.M.K.); susan.morton1@my.jcu.edu.au (S.K.M.); lijiaze@hotmail.com (J.L.); 2 Australian Institute of Tropical Health and Medicine, James Cook University, Townsville, QLD 4811, Australia; 3 Department of Vascular and Endovascular Surgery, Townsville University Hospital, Townsville, QLD 4811, Australia

**Keywords:** abdominal aortic aneurysm, aneurysm rupture, rupture risk, aortic stiffness, peak wall stress, preclinical imaging

## Abstract

Abdominal aortic aneurysm (AAA) rupture is an important cause of death in older adults. In clinical practice, the most established predictor of AAA rupture is maximum AAA diameter. Aortic diameter is commonly used to assess AAA severity in mouse models studies. AAA rupture occurs when the stress (force per unit area) on the aneurysm wall exceeds wall strength. Previous research suggests that aortic wall structure and strength, biomechanical forces on the aorta and cellular and proteolytic composition of the AAA wall influence the risk of AAA rupture. Mouse models offer an opportunity to study the association of these factors with AAA rupture in a way not currently possible in patients. Such studies could provide data to support the use of novel surrogate markers of AAA rupture in patients. In this review, the currently available mouse models of AAA and their relevance to the study of AAA rupture are discussed. The review highlights the limitations of mouse models and suggests novel approaches that could be incorporated in future experimental AAA studies to generate clinically relevant results.

## 1. Introduction

Abdominal aortic aneurysm (AAA) is a chronic degenerative disease that results in the focal expansion of all layers of the abdominal aortic wall. The risk factors for AAA include older age, male sex, smoking, hypertension, dyslipidemia, other cardiovascular diseases, and family history of AAA. AAA is usually diagnosed when an asymptomatic person undergoes incidental imaging or ultrasound screening, which identifies a focal abdominal aortic dilatation with a diameter ≥30 mm [1,2,3]. The main complication of AAA is rupture, which is estimated to be responsible for approximately 200,000 deaths per year worldwide [4]. The most established predictor of AAA rupture is maximum aortic diameter [5]. Current guidelines recommend that small asymptomatic AAAs (<50 mm in women and <55 mm in men) are managed by interval surveillance imaging. Large or symptomatic AAAs are usually considered for surgical repair by either an open or endovascular operation [6,7].

AAA rupture is believed to occur when the hemodynamic forces from pulsatile blood flow exceed the strength of the AAA wall. Even though the exact mechanisms of AAA rupture are unknown, several pathological processes, including infiltration of inflammatory cells including T regulatory cells [8], proteolytic degradation of the extracellular matrix (ECM), and neovascularization have been implicated in the process. These biological processes are believed to cause elastin degradation and alter collagen composition that compromises the strength and elasticity of the aorta [9,10]. It is still unclear to what extent the different biological processes individually contribute to the mechanical changes and eventual rupture of the aortic wall.

## 2. Clinical Risk Factors for AAA Rupture

Studying the natural history of AAA in patients is difficult since in most countries people with large asymptomatic AAAs undergo elective surgical repair. Furthermore, post-mortems are rarely performed and thus the exact cause of death in older people in frequently unknown. Studies examining risk factors for AAA rupture are mainly limited to investigations of people with small AAAs undergo imaging surveillance, where the rupture incidence is very low (approximately 1% per year) [11,12]. There have also been a few studies of people with large AAAs whom have been deemed unsuitable for AAA repair or who have refused AAA repair. The largest being a study involving 47 Veterans Affairs medical centres in the USA [5].

### 2.1. AAA Diameter

Abdominal aortic diameter is the best-known predictor of AAA rupture and is used to guide clinical practice [6,7]. In a study of 198 veterans with large AAAs in which elective AAA repair was not planned, due to co-morbidities or patient refusal, the estimated one year risk of AAA rupture was 9.4% for 55 to 59 mm, 10.2% for 60 to 69 mm, and 32.5% for ≥70 mm diameter AAAs [5]. A more recent systematic review which pooled data from 11 studies involving 1,514 patients, estimated substantially smaller one-year rupture rates of 3.5%, 4.1%, and 6.3% for AAAs with an initial AAA diameter of 55–60 mm, 61–70 mm, and >70 mm, respectively [13]. It was noted that most of these unfit patients died of causes other than AAA rupture [13].

### 2.2. AAA Growth Rate

Larger AAAs grow at a faster rate [14,15]. A previous systematic review reported that a 10 mm larger initial AAA diameter was associated with a mean increase in annual growth rate of 1.6 mm [15]. Due to the strong associations of AAA diameter with both rupture-risk and AAA growth, identifying whether there is an independent association of AAA growth with AAA rupture-risk is difficult. In the Veterans Affairs study discussed above, faster AAA growth was associated with a greater risk of AAA rupture in unadjusted analyses, but this association was lost after adjusting for initial AAA diameter [5]. Overall, there does not appear to be convincing evidence that faster AAA growth is associated with a greater risk of AAA rupture independent of AAA diameter. This may be partly due to the difficulties of accurately measuring small changes in AAA diameter in clinical practice [16].

### 2.3. Female Sex

Rupture of small AAAs under surveillance appears to be about 4 times more likely to occur in females than males based on data from the largest meta-analysis of individual patient data [12,17,18]. At a similar age, healthy males typically have a much larger aortic diameter compared to females [18]. This difference in normal aortic diameter in women and men most likely explains the higher risk of rupture in women compared to men with similar size AAAs. In other words, women with similar size AAAs as men likely have more severe later stage disease. As a result of these sex differences, current guidelines recommend surgical repair is considered at a lower AAA diameter (≥50 mm) in women than men (≥55 mm) [6,7]. 

### 2.4. Smoking

A history of current smoking has been independently associated with a 2-fold increased risk of AAA rupture, relative to a history of former or never smoking in people with small AAAs [12]. Studies in mouse models have attempted to examine the mechanisms by which smoking promotes AAA rupture [19]. Tobacco smoke is a complex mixture of over 5000 different chemicals, therefore pinpointing the exact mechanism is difficult. It has been suggested that smoke exposure primes leukocytes to promote aortic degradation. Cigarette smoke also activates endothelial cells to express adhesion molecules and chemokines promoting leukocyte infiltration into the aortic wall [20]. In one mouse study, adoptive transfer of leukocytes from mice exposed to cigarette smoke promoted induction of larger aneurysms in an elastase model [21]. Benzo(a)pyrene, a constituent of cigarette smoke, has been reported to increase aortic macrophage infiltration, active nuclear factor-κB (NF-κB) and promote rupture-prone aneurysms within the angiotensin II (AngII)-infusion mouse model [22]. The complex nature of cigarette smoke and the uncertainty about translating findings from rodent models to humans means that the exact mechanisms responsible for the association of smoking with an increased risk of AAA rupture remain unclear.

### 2.5. Hypertension

After adjusting for AAA diameter, a 10 mmHg higher mean arterial blood pressure has been associated with a 1.3-fold higher risk of small AAA rupture [12]. A pulse pressure increase of 10 mmHg was independently associated with a smaller but significant 1.1-fold increased risk of small AAA rupture [12]. Since blood pressure has an important influence on the hemodynamic forces exerted on the aorta, these findings support the importance of biomechanical forces in stimulating AAA rupture. The next section focuses on the role of biomechanical forces in AAA rupture.

## 3. Biomechanics Forces and AAA Rupture

The biomechanical properties of the aortic wall are believed to depend upon the composition and structure of the aortic ECM. Aortic compliance is a measure of the change in the internal volume of the vessel, brought on by a change in intraluminal pressure, and is largely determined by the elastic components of the aortic wall [23,24]. 

### 3.1. Aortic Compliance

The aortic wall is considered compliant, or distensible, when small changes in intraluminal pressure lead to a large change in volume [24]. A prospective study of 210 people with an AAA reported that those with greater aortic distensibility had a significantly higher risk of aneurysm rupture (hazards ratio: 1.38; 95% CI: 1.08–1.78; *p* = 0.011) [25]. High distensibility was independently associated with aneurysm rupture after adjusting for other risk factors, such as age, sex, AAA diameter, and blood pressure [25]. Accurate ways to measure aortic distensibility in routine practice are needed.

### 3.2. Peak Wall Stress 

Peak wall stress (PWS) is an estimation of the mechanical stress on the AAA wall that arises perpendicular to the blood flow. Using finite element analysis (FEA), PWS can be estimated non-invasively from computer tomography (CT) scans. A systematic review and meta-analysis which collated published data from 9 independent studies, found that PWS was significantly higher in patients with a symptomatic or ruptured AAA compared to patients with an intact AAA (*n* = 144 and 204, respectively; *p* < 0.001) [26]. In many of the included studies, the ruptured AAAs had larger diameter than the intact AAAs. The independent association of high PWS with aneurysm rupture remains under investigation. In one study, PWS had increased sensitivity (94% vs. 81%), accuracy (85% vs. 73%) and specificity (81% vs. 70%) as a predictor of rupture-risk relative to maximum diameter [27]. A further systematic review highlighted a number of methodological weaknesses of prior studies [28]. There is therefore remaining uncertainty about the value of measuring PWS to predict AAA rupture [28].

### 3.3. Aortic Calcification

The association of micro- and macro-calcification of the aortic wall with AAA rupture is controversial. In one study, higher aortic calcification score measured from CT imaging was associated with greater risk of AAA rupture [29]. Studies of AAA tissue samples have suggested that the junction of calcified and non-calcified tissue may represent a weak site [10]. Micro-calcification has been associated with an increased risk of rupture of atherosclerotic plaques [30] and also an increase rate of AAA expansion [31]. In a recent mouse study, application of hydroxyapatite (a main component of micro-calcification) promoted AngII-induced AAA formation [31]. Larger studies using accurate methods to assess aortic wall calcification are needed to resolve the role of calcification in AAA rupture.

## 4. Mouse Models of AAA

Multiple different mouse models of AAA have been described, but many of these do not demonstrate aneurysm rupture (Table 1) [32,33]. Mouse models that do illustrate aortic rupture are described below. Prior to starting any animal experiment, careful consideration has to be given to animal welfare. Before starting any animal study, particularly those where aneurysm rupture is likely, it is vital researchers obtain advice from qualified veterinarians about how to plan the investigation so as to minimize any animal suffering.

### 4.1. Angiotensin II (AngII) Infusion Model

**Figure 1:**
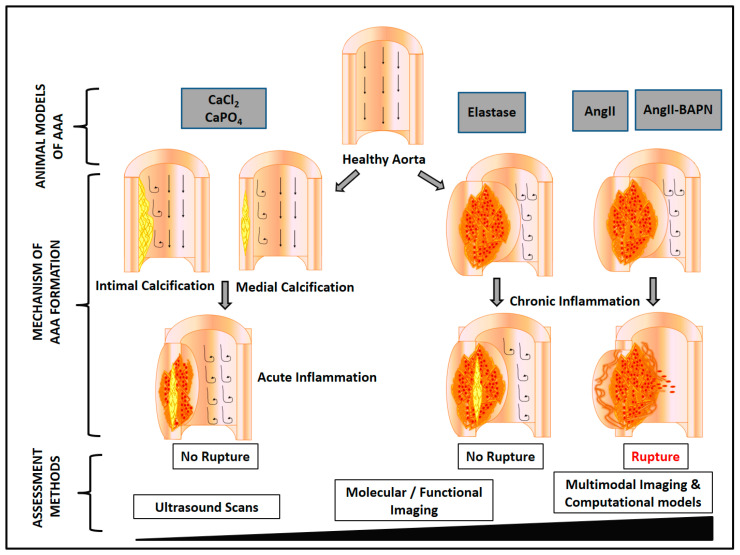
Methods to induce AAA in mice. Aortic application of calcium chloride (CaCl_2_) or calcium phosphate (CaPO_4_) promotes aneurysm formation but not rupture. In the elastase model, disruption of elastin layers promotes monocyte recruitment and subsequent release of interleukin-6 and matrix metalloproteinases leading to ECM degradation. Aneurysm rupture is rarely reported. In AngII-induced aneurysms, aortic inflammation promotes ECM degradation, vascular smooth muscle cell apoptosis, aortic dissection, intramural thrombus formation and rupture. In the AngII-BAPN model, ECM degeneration and mural dissections are more advanced, associated with a higher rate of aortic rupture. The resolution of the methods used to assess outcomes in these models have increased considerably. High resolution ultrasound and multimodal functional imaging allow accurate monitoring of aortic diameter and may enable molecular changes to be related to aortic rupture in vivo. Abbreviations: Ang II, angiotensin II; BAPN, β-aminopropionitrile monofumarate; CaCl_2_, calcium chloride, CaPO_4_, calcium phosphate; ECM, extracellular matrix.

This is the most widely reported animal model of AAA [75,76,77]. Mini osmotic pumps that are subcutaneously implanted deliver AngII (usually 1000 ng/kg/min). In susceptible mice, such as those which are apolipoprotein E deficient (*ApoE^-/-^*), AngII induces aortic dissection and aneurysm development in the supra-renal and thoracic aorta. This is in contrast to AAA in humans, which are most frequently located in the infra-renal aorta. About 15% to 30% of mice develop abdominal or thoracic aortic rupture. A recent study by Trachet et al., using synchrotron-based ultra-high-resolution phase contrast X-Ray tomographic microscopy (PCXTM) reported novel details of aneurysm rupture in the AngII-*ApoE^-/-^* model [78]. They identified the presence of a tear near the coeliac artery that led to rupture of the tunica media near the ostium of small supra-renal side branches in some mice. They proposed that a rupture in the tunica media led to an intra-mural haematoma or intra-mural thrombus (IMT) and dissection of the tunica adventitia in the supra-renal aorta. The AngII-*ApoE^-/-^* model may therefore be a better model of aortic dissection-related than fusiform AAA-related rupture [78]. The AngII-*ApoE^-/-^* model has IMT rather than intra-luminal thrombus (ILT) which is found in most human AAAs (Figure 1).

A number of variations in the AngII model that lead to increased aortic rupture have been described. These include injection of anti-transforming growth factor (TGF)-β antibody [66,67] and administration of kinin agonists or the lysyl oxidase inhibitor β-aminopropionitrile monofumarate (BAPN) [61,79,80] (Table 1). AngII-induced aortic rupture is enhanced markedly by TGF-β neutralization [66,67]. Prolonged infusion of AngII has been reported to promote continued aortic expansion [81]. Combining an extended period of AngII infusion and TGF-β neutralization leads to a high incidence of aortic rupture [82]. Lysyl oxidase mediates cross-linking of tropo-elastin monomers to form an insoluble functional elastin polymer and cross-linking of tropo-collagen to form collagen fibrils. Disruption of lysyl oxidase leads to weakness of the aortic wall [83,84]. In the AngII-BAPN model, AngII infusion is combined with oral administration of BAPN. This model has some features of human AAA, including aortic neutrophil infiltration and overexpression of matrix metalloproteinase (MMP)-9. The incidence of AAA in this model has been reported to be 100% [61,85]. AAA incidence has been reported to reduce to 40% following the administration of an MMP inhibitor [61]. The AngII-BAPN model has been reported to have a higher incidence of aortic rupture compared to other mouse models [61,67,79]. A recent study analysed aortic ruptures in this model using propagation-based phase-contrast synchrotron imaging [61]. This imaging technique allowed individual elastic layer tracking as a means to precisely detect inter-lamellar hematoma formation. The study suggested that this model illustrated both ILT and IMT (Figure 1). 

Another modification of the AngII-*ApoE^-/-^* AAA model involved peri-aortic application of leptin [65]. Aneurysms in this model have some classical features of human AAA, such as ECM degeneration, MMP up-regulation, and macrophage infiltration. Aortic dissections and hematomas have been reported in this model but not aortic rupture [65] (Table 1). This most likely reflects the limited study of this model, since AngII has been widely reported to induce aortic rupture. 

### 4.2. Elastase Model

Intraluminal infusion or adventitial application of elastase to the infra-renal aorta stimulates AAA formation in rodents [72,86]. In the intra-luminal elastase model, AAAs are created by continuously perfusing porcine pancreatic elastase (PPE) into the aortic lumen. The aneurysms created are generally small and do not rupture. Endothelial damage and destruction of the elastic fibres is followed by the infiltration of inflammatory cells. The aortic diameter expansion achieved in this method is about 2-fold after 1 to 2 weeks. Creation of this model requires advanced surgical skills meaning there is a substantial learning curve. As a result of these technical challenges, the model has been modified to externally apply, rather than intraluminal infuse, PPE to the infra-renal aorta (for 10 to 40 min) [87,88,89,90]. This is technically simpler, but as with the classical intra-luminal elastase model, AAA rupture is rarely seen (Table 1, Figure 1).

A number of modifications of the adventitial elastase model have been reported to promote the development of rupture-prone AAAs. Combining adventitial application of elastase with systemic blockade of TGF-β activity has been reported to induce rapid AAA rupture [72,73]. Aortas from this model have significantly enhanced elastin degradation. Synchrotron-based ultrahigh resolution imaging showed extensive aortic elastin degradation and the presence of a large amount of ILT [72]. Even though this model is ideal for studying aortic rupture, the survival rate of mice receiving elastase and anti-TGF-β is low, posing animal welfare concerns [72,73]. 

Peri-adventitial aortic elastase application in combination with oral BAPN fumarate (0.2%) has been reported to promote large AAAs in C57BL/6J mice [62,74]. This combination of interventions has been reported to promote aortic diameter expansion to up to 364% of the original size. Histological assessment showed extensive elastin fiber degradation. This model has also been reported to illustrate aortic rupture [62,74]. 

In order to better simulate human fusiform, infra-renal AAA improvements are needed in currently available mouse models. Human fusiform AAAs form over decades and rupture occurs at a late stage after prolonged aortic expansion. Most currently available mouse models simulate rupture associated with aortic dissection. It is possible that by modifying the current models a more prolonged aneurysm progression stage may be simulated. Reducing the AngII dose and increasing the infusion time may better reflect the slow growing human AAA. Prolonged AngII infusion was shown to induce aneurysm growth progressively in an 84-day study [81]. Some of the novel combination models, such as the adventitial elastase and BAPN model hold promise for better simulating human AAA.

## 5. Use of New Imaging Methods to Study AAA Rupture

Use of novel imaging methods could advance the prediction and understanding of AAA rupture.

### 5.1. High-Resolution Ultrasound to Study AAA Rupture

**Figure 2:**
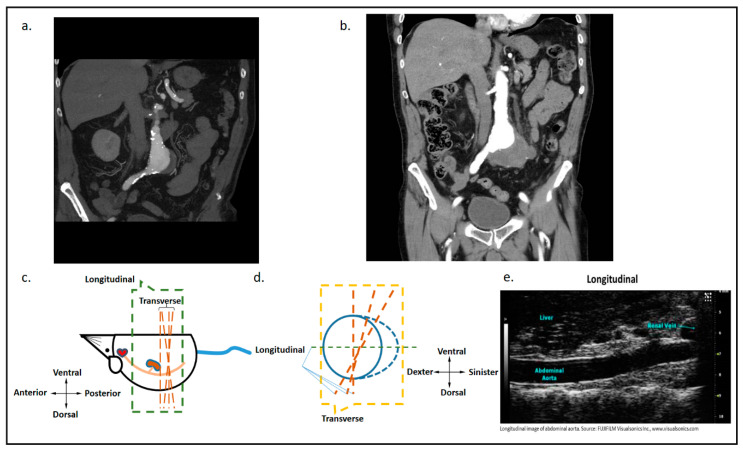
Coronal images of computed tomographic angiograms of a patient with an AAA and visualisation of an AAA in a mouse model. (**a**) The image shows a coronal slice of a computed tomographic angiogram of an intact AAA measuring a maximum diameter of approximately 47 mm. (**b**) One year later the, computed tomographic angiogram showed a retroperitoneal leak from the AAA. The patient underwent successful emergency endovascular aneurysm repair. (**c**) An illustration of the longitudinal and transverse planes when scanning a mouse aorta using ultrasound in the longitudinal axis. (**d**) An illustration of longitudinal and transverse planes when scanning a mouse aorta using ultrasound in the transverse axis. (**e**) A longitudinal plane image of a mouse aorta using micro high-resolution ultrasound. Image source: application brief: abdominal aortic aneurysm, Fujifilm Visualsonics Inc. Permission was obtained to reproduce the image in this manuscript.

Ultrasound is the primary technique used in AAA screening and assessment due to its high accessibility, ease of operation, and its capability for real-time non-invasive in vivo imaging (Figure 2). Ex vivo morphometry assessment of AAA has a number of limitations, such as the inability to measure aortic diameter at maximum expansion. Ultrasound, however, allows accurate in vivo assessment of AAA. The recent introduction of high-resolution ultrasound technology such as the Visualsonic Vevo system has greatly improved the preclinical imaging of mouse models. The major advantage of high-resolution ultrasound over conventional ultrasound is high image quality. Compared to the 20 kHz frequency probe used in conventional ultrasound, high-resolution ultrasound often uses 20 to 100 mHz frequency probes. High resolution ultrasound has a resolution of up to 30 µm. Diameter measurements can be performed reproducibly. A quantitative variability study by Sampson et al. [91], reported low repeat measurement variability for mouse iliac, infra-renal, right renal, supra-renal, and thoracic aorta diameters. Both transverse imaging (short-axis) and longitudinal imaging (long-axis) of the aorta can be performed (Figure 2). The Visualsonic Vevo 770 further allows 3D data construction, which enables better visualization of the aneurysm from different angles and planes. Other functional modules of high-resolution ultrasound, such as color doppler mode and electrocardiography, permit the assessment of additional parameters potentially assisting the evaluation of aneurysm rupture-risk. Ultrasound has also been used to assess fluid dynamics (flow pattern, flow rate and aortic volume), aortic wall dynamics (displacement ratio, wall thickness and radial wall velocity), circumferential strain, wall stiffness and vascular calcification. Further studies are required to establish the feasibility of assessing these outcomes in relation to AAA rupture. 

In a novel preclinical study, a biotechnological theranostic approach was used in the AngII-ApoE^-/-^ mouse model [92]. MicroRNA-126 was previously shown to downregulate vascular cell adhesion molecule-1 (VCAM-1) expression in endothelial cells and to exert anti-inflammatory effects by reduced leukocyte adhesion. The investigators used ultrasound-microbubbles coupled with VCAM-1-targeted antibodies (scFvmVCAM-1) and microRNA-126 mimic theranostic microbubbles (TargMB-M126) [92]. They showed that TargMB-M126 inhibited AngII-induced AAA. This targeted approach to delivering interventions holds great promise to selectively deliver effective treatments in patients.

Recently, a 4D-ultrasound imaging approach was used to evaluate geometric and biomechanical parameters in the BAPN-Elastase model [74]. 4D-ultrasound uses serial cine loops of 2D-B-mode images over a prescribed distance to visualize volume changes throughout the cardiac cycle. This allowed the estimation of vessel tortuosity, surface area and biomechanical metrics, such as arterial stress and circumferential strain. The investigators failed to explore the role of ILT in AAA rupture. This would be valuable to study in the AngII-BAPN model in the future.

### 5.2. Functional and Molecular Imaging

Functional imaging using biomarkers or molecular tracer agents in order to assess molecular changes in vivo. A combination of morphological imaging techniques, such as CT or magnetic resonance imaging (MRI), with functional imaging provide a means to study AAA rupture-related mechanisms in real-time in vivo. Inflammatory cells present in the AAA wall are believed to secrete proteases, such as MMPs, that degrade aortic ECM. Molecular-MRI and positron emission tomography–computed tomography (PET-CT) using nanoparticles labeled with isotopes targeting collagen, monocytes/macrophages, and MMPs could provide a means to monitor the risk of AAA rupture. 

Molecular-MRI has been used to assess inflammatory and elastase activity using a macrophage-specific iron oxide-based probe (Ferumoxytol) and an elastin-specific Gadolinium-based probe in the AngII-ApoE^-/-^ model [93]. The study reported that this imaging agreed with ex vivo histology and was able to predict AAA rupture [93]. 

Another novel functional imaging approach is ultra-small superparamagnetic particles of iron oxide (USPIO)-enhanced MRI that detects cellular inflammation. In a prospective multicentre open-label cohort study, patients (*n* = 342) were classified by relative USPIO uptake and monitored with serial ultrasound [94,95]. USPIO uptake was not independently associated AAA growth or rupture risk, suggested that this approach may not be of value in clinical practice.

PET-CT is commonly used in the assessment of cancer. PET-CT has been reported to successfully localize and quantify monocytes/macrophages in AAAs in mouse models and patients. Nahrendorf et al. reported successfully quantifying AAA macrophage content in a mouse model using PET-CT and macrophage-targeted nanoparticles labeled with Fluorine-18 [96]. Fluorine-18 fluorodeoxyglucose ((18F)-FDG) is a commonly used radiotracer in clinical practice which is taken up at sites where there is high metabolic activity, e.g., inflammation. (18F)-FDG uptake has been correlated with the development and progression of both abdominal and thoracic aortic aneurysms in clinical and preclinical studies [97,98,99]. A previous systematic review summarised prior studies of inflammatory imaging biomarkers and their association with AAA growth and rupture [100]. The review suggested that there was no convincing evidence that (18F)-FDG or USPIO uptake were reliably predictive of AAA growth or rupture. 

In contrast, the sodium fluoride imaging of AAA (SoFIA3) trial suggested that Fluorine-18 sodium fluoride uptake, which is reflective of micro-calcification, was predictive of AAA growth, requirement for repair or rupture [101]. The SoFIA3 trial was a prospective case-control (*n* = 20/group) and longitudinal cohort (*n* = 72) study of patients with AAA. The imaging quantified active calcification using Fluorine-18-labeled NaF (18F-NaF) PET-CT, CT angiography, and calcium scores. The clinical endpoints assessed were AAA growth and a combination of requirement for AAA repair or AAA rupture. AAA patients had significantly increased 18F-NaF uptake compared to controls. Patients with 18F-NaF uptake had a three-fold higher risk of AAA repair or rupture, when compared to controls [101]. This study provides the first proof-of-concept data for PET-CT as a potential imaging assessment for AAA risk-stratification. A recent pre-clinical study using the AngII-infusion AAA model reported that the uptake of the cell proliferation radiotracer, Fluorine-18-fluorothymidine ((18F)FLT) is increased during the active growth phase of the AAA [102]. Addition of such novel radiotracer agents to PET-CT could enhance the ability to identify which AAAs require surgical repair.

Myeloperoxidase (MPO) is expressed in inflammatory cells identified within AAA samples. Thus molecular imaging targeting MPO expressed within inflammatory cells could be another, not yet reported, method of assessing AAA rupture risk. The technique of dual-target PET/MRI of transglutaminase factor XIII (FXIII) and MPO activity has been studied within infarcted hearts of *ApoE^-/-^* mice [103]. The Fluorine-18-labeled PET agent ((18)F-FXIII) was used to enhance resolution and MPO-gadolinium was used to image MPO-rich inflammatory leukocytes. This imaging approach was found to be a sensitive method to detect the effects of intravenous therapeutics, such as nanoparticles, suggesting the potential value for AAA imaging.

Klink et al. used gadolinium-labelled paramagnetic/fluorescent lipid micelle nanoparticles functionalized with a collagen-binding protein (CNA-35) to measure collagen content in mouse aneurysmal walls [104]. MRI of aortic walls in these mice revealed an association between decreased collagen content and increased risk of rupture. Though not yet evaluated in AAA studies, molecular imaging of MMP expression has also been performed. Near infrared (Near-IR) is another emerging molecular imaging technique that enables investigation of vascular protein composition including collagen and elastin. A study conducted by Urbas et al. in 2003 used ex vivo aortic samples to discover distinct spectrums of each of the main collagens (I-IV) and elastins [105]. A relation between collagen to elastin ratio and the dose of the AAA inducting agent was also reported in this study. Since both collagen and elastin are important aortic wall matrix proteins, the ability to detect and quantify these proteins in vivo may be useful in assessing wall integrity. Further development of these molecular imaging techniques may offer clinically useful mean to assess AAA rupture risk.

## 6. The Future of AAA Imaging and Rupture Prediction

Imaging methods which combine a range of molecular and anatomical assessments are being developed. PET-CT camera systems which incorporate MRI are being studied for example [106]. PET-MRI hybrid imaging has been reported to reduce the scan time and seamlessly co-registers images acquired through the different modalities and permits synchronized evaluation of multiple imaging probes [101,103,107,108]. Such combination imaging offers immense potential to better understand the progress of disease in vivo and may provide a means to better predict AAA rupture and develop specific drug therapies.

ILT is a common feature of human AAA [109]. ILT obtained during open AAA repair have large numbers of activated platelets and leukocytes [110]. A study using the elastase-perfused rat model assessed platelet activation using 99mTc-Annexin-V staining with single-photon emission CT (SPECT) imaging [110]. The study reported a 5-fold increase in target-to-background ratio in aortic areas where there was a high number of activated platelets as compared to the normal aortic wall. Similarly, when the P-selectin-specific targeting agent 99mTc-fucoidan was used, activated platelets were detected with a median target-to-background ratio of 3.6 in the elastase rats [111]. The studies highlight the potential of studying thrombus-related cellular activity in AAA. 

Results of a recent systematic review highlight that the role of ILT in AAA rupture is controversial [112]. If the biological activity of ILT can be better quantified, a clearer understanding of the role of ILT in AAA progression may become evident. A recent computational modelling study investigated AAA evolution over time [113]. The authors concluded that when modelling AAA growth, the choice of tissue growth kinematics is of critical importance. Further interdisciplinary experimental work is required to develop and validate aortic tissue adaption models. 

## 7. Conclusions

AAA diameter remains the most established measure of AAA rupture risk. The explosion of molecular imaging techniques offers great promise in better monitoring disease progression in vivo. Mouse models offer unique ways to study AAA rupture although their translational relevance is still uncertain. By using high-resolution ultrasound and functional and molecular imaging techniques, new assessments, such as fluid dynamics, aortic wall dynamics, circumferential strain, wall stiffness, vascular calcification, vessel wall inflammation, and ECM degradation can be performed to estimate AAA rupture risk. It is clear that each preclinical model has its own unique strength and weakness and among the various rodent AAA models showing rupture, the combined BAPN-AngII mouse model might be a more appropriate preclinical AAA model due to the presentation of ILT and associated rupture. It remains uncertain which, if any, imaging approach will replace CT and ultrasound routinely used in clinical practice today. Further validation of new imaging techniques in clinically relevant AAA mouse models will help develop approaches that can improve rupture risk stratification in clinical practice. Use of novel molecular imaging and pre-clinical animal models is expected to improve understanding of the biological mechanisms involved in AAA rupture.

## Figures and Tables

**Table 1:** Examples of commonly used mouse models of abdominal aortic aneurysm (AAA). Key histological and pathological features of each model and their rupture characteristics when compared to human AAA are outlined.

Model	Key Features	Rupture Characteristics	Reference
***Genetic***			
*Timp-1 ^-/-^* Deficient in TIMP-1 gene	Proteolysis ECM degradation Inflammation Aneurysms in both the thoracic and abdominal regions of the aorta	No ruptures reported	[34,35,36,37,38,39]
*ApoE ^-/-^* Deficient in ApoE gene	Dyslipidemia Atherosclerosis Require other AAA induction agents such as high fat diet or chemical induction	No ruptures reported in genetic deficiency alone	[40,41,42,43,44,45]
Blotchy and *Lox ^-/-^*	Lack crosslinks between elastin and collagen fibers Elastin fragmentation VSMC apoptosis Aneurysms along the full length of the aorta	No ruptures reported in most studies One study reports that males do rupture and females do after hydrocortisone treatment	[46,47,48,49] [31]
Overexpression of Renin and Angiotensinogen	Hypertension, Inflammation Medial degeneration Require high salt intake Aneurysms and ruptures Aneurysms in the thoracic and abdominal regions of the aorta	No ruptures reported	[50,51]
***Chemical***			
*CaCl_2_* Painting solubilised CaCl_2_ on the exposed IRA	Aortic calcification Medial degeneration Inflammatory cells infiltration	No ruptures reported	[32,52,53,54]
*CaPO_4_* Painting solubilised CaPO_4_ on the exposed IRA	Aortic calcification Medial degeneration Inflammatory cells infiltration	No ruptures reported	[55,56]
*AngII infusion* Delivering AngII through subcutaneously implanted osmotic pumps	Acute aortic dissections, aneurysms and ruptures Upregulation of chemokines and pro-inflammatory cytokines Leukocyte infiltration ECM degeneration VSMC apoptosis	Ruptures often occur within first week of AngII infusion in the arch, thoracic and SRA regions	[33,57,58,59,60]
*AngII + BAPN* AngII infusion to mice that had received BAPN	Leads to higher incidence of AAA Medial ECM degeneration VSMC apoptosis Dissections are common with presence of ILT and IMT	Fatal medial ruptures in the IRA	[61,62,63,64]
*AngII + Leptin* Peri-aortic application of Leptin in the *ApoE^-/-^* mouse combined with AngII infusion	ECM degeneration MMP up-regulation Macrophage infiltration Dissections and IMT are present	No ruptures reported	[65]
*AngII + anti-TGF-β antibody*	Aortic dissection Enhanced monocyte infiltration Increased MMP-12 activity	Increased rupture in both the ascending aorta and SRA	[66,67]
*Elastase perfusion* Elastase is delivered through a catheter placed in the IRA	Inflammation Medial degeneration and ILT present	No ruptures reported	[68,69,70,71]
*Elastase + TGF-β activity Blocking* Combined adventitial application of Elastase and neutralizing TGF-β activity by mouse monoclonal antibody	Enhanced elastin degradation Ongoing inflammation Presence of large amount of ILT	Fatal rupture in IRA	[72,73]
*Elastase + BAPN* Combined adventitial application of Elastase on mice that had received BAPN	Higher AAA incidence Medial ECM degeneration VSMC apoptosis ILT presence	Fatal rupture in IRA	[62,74]

Abbreviations: AngII, angiotensin II; ApoE, apolipoprotein E; BAPN, β-aminopropionitrile monofumarate; CaCl_2_, calcium chloride, CaPO_4_, calcium phosphate; ECM, extracellular matrix; ILT, intra-luminal thrombus; IMT, intra-mural thrombus; IRA, infrarenal aorta; Lox, lysyl oxidase; MMP, matrix metalloproteinase; SRA, suprarenal aorta; TIMP, tissue inhibitor of metalloproteinase; VSMC, vascular smooth muscle cells.

## Abbreviations

AAA	Abdominal aortic aneurysm
ApoE	Apolipoprotein E
AngII	Angiotensin II
CT	Computer tomography
ECM	Extracellular matrix
ILT	Intra-luminal thrombus
IMT	Intra-mural thrombus
MAP	Mean arterial pressure
MMP	Matrix metalloproteinase
PWS	Peak wall stress
VSMC	Vascular smooth muscle cells
WSS	Wall shear stress
